# Translocational renal cell carcinoma (t(6;11)(p21;q12) with transcription factor EB (TFEB) amplification and an integrated precision approach: a case report

**DOI:** 10.1186/s13256-015-0749-7

**Published:** 2015-12-09

**Authors:** Wolfgang Lilleby, Ljiljana Vlatkovic, Leonardo A. Meza-Zepeda, Mona-Elisabeth Revheim, Eivind Hovig

**Affiliations:** 1 Department of Oncology, Oslo University Hospital, Nydalen, Postboks 4950, 0424 Oslo, Norway; 2 Department of Pathology, Oslo University Hospital, Nydalen, Postboks 4950, 0424 Oslo, Norway; 3 Department of Radiology and Nuclear Medicine, Oslo University Hospital, Nydalen, Postboks 4950, 0424 Oslo, Norway; 4 Department of Tumor Biology, Institute for Cancear Research, Oslo, Oslo University Hospital, Nydalen, Postboks 4950, 0424 Oslo, Norway; 5 Department of Core Facilities, Institute for Cancer Research, Oslo University Hospital, Nydalen, Postboks 4950, 0424 Oslo, Norway; 6 Department of Cancer Genetics and Informatics, University of Oslo, Oslo University Hospital, Nydalen, Postboks 4950, 0424 Oslo, Norway; 7 Department of Informatics, University of Oslo, Blindern, Postboks 1080, 0316 Oslo, Norway

**Keywords:** Autophagy, Gene signature, Translocation renal cell carcinoma

## Abstract

**Introduction:**

Renal cell carcinoma with the distinct type of t(6;11)(p21;q12) translocation (transcription factor EB) is a rare neoplasm. In the present case study, we show for the first time an autophagy signature in a patient with transcription factor EB renal cell carcinoma. We attempted to characterize the mutational and expressional features of a t(6;11)(p21;q12) renal cell carcinoma, in an effort to address the potential for molecular guidance of personalized medical decision for a case in this renal cell carcinoma category.

**Case presentation:**

We report the case of a 42-year-old white man who had a late relapse of his renal cell carcinoma. The first diagnosis of clear cell renal carcinoma was derived from a histological examination; analyzing the metastasis and going back to the primary tumor it turned out to be a transcription factor EB-renal cell carcinoma. The treatment plan included local radiation and systemic therapy. As part of the multimodal approach, tumor samples for genetic assessment were obtained. However, there is no recommended standard therapy for transcription factor EB-renal cell carcinoma. Despite four lines of medical treatment with targeted therapy and one checkpoint inhibitor, all attempts to prolong the patient’s survival failed.

**Conclusions:**

During the course of this unusual disease, we gained insights which, to the best of our knowledge, were unknown before in the expression of the gene signature linked to autophagy. This might in part explain the resistance to conventional targeted therapy acknowledged in our patient.

**Electronic supplementary material:**

The online version of this article (doi:10.1186/s13256-015-0749-7) contains supplementary material, which is available to authorized users.

## Introduction

The translocation involves on chromosome 6 the *MITF* transcription factor family member transcription factor EB (TFEB), and on chromosome 11 the metastasis-associated lung adenocarcinoma transcript 1 (MALAT1). It has previously been shown that the TFEB transcription factor is involved in the regulation of autophagy (see [1] for a review of the role of TFEB in this setting). Sardiello *et al.* defined an autophagy gene network regulating lysosomal biogenesis and function through a systematic study of TFEB upregulation [2]. Indirectly, these features can be observed immunohistochemically when tumor cells express cathepsin K in the cytoplasm [3]. This is a consequence of the dysregulation induced by the translocation, as TFEB controls cathepsin K expression. TFEB protein is highly expressed in osteoclasts and TFEB is linked to RANKL activity [4].

Among the published cases of this pathologic translocation variant, it appears that the therapeutic targets against common renal cell carcinoma (RCC) may not be effective against translocation type RCC, possibly due to distinct molecular alterations of these carcinomas [5].

## Case presentation

A 42-year-old white man had a history of kidney cancer from 2005. At that time, his right kidney was removed, and RCC was concluded on the basis of histology. The disease was staged as T3apN0M0 (TNM-Union for International Cancer Control, TNM-UICC, 2010). The cancer was regarded as localized and no further investigation was undertaken at the time. In the winter of 2013, the patient consulted his general practitioner for increasing right-sided lateral chest pain. Computed tomography (CT) of his thorax revealed a lateral mass destructing his third rib, without penetrating through the pleural cavity. Several biopsies were obtained and the tumor was confirmed as clear cell RCC. However, the pathologist responsible had doubts about the correct histology and consulted experts in the field for a second opinion abroad. Techniques including Sanger sequencing of the candidate translocation region and fluorescence *in situ* hybridization (FISH) confirmed a translocation including TFEB (t6;11), which is diagnostic of RCC. Immunohistochemistry was positive for cathepsin K and Melan-A, which supported the diagnosis.

**Fig. 1:**
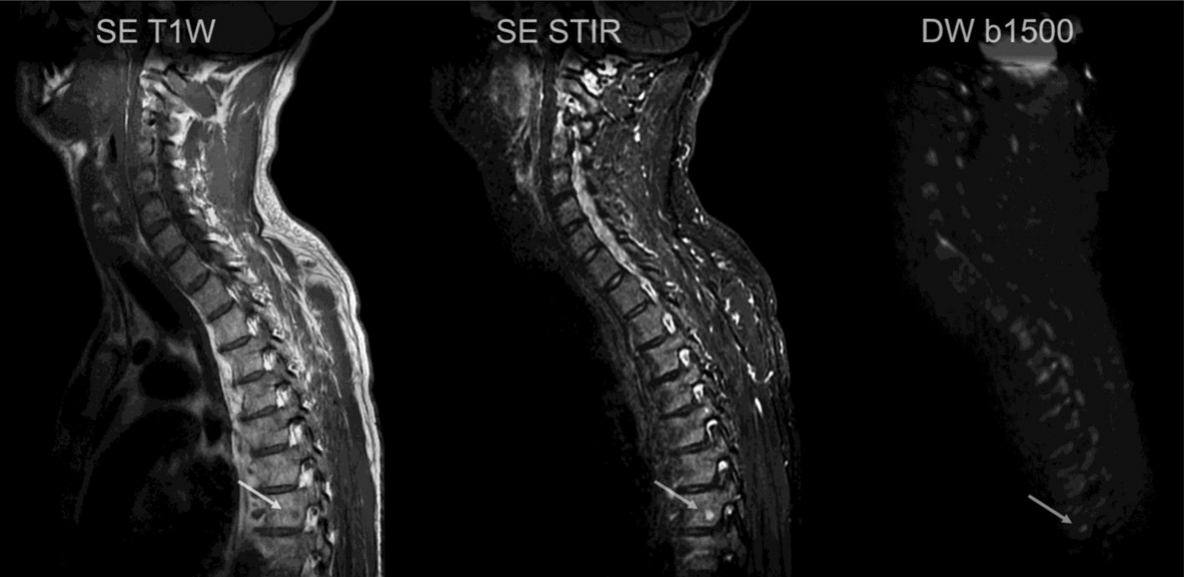
Magnetic resonance imaging visualizing metastatic spine lesion (*arrow*)

Further investigation of the patient with magnetic resonance imaging (MRI; Fig. 1) of his vertebral column showed small lesions in his lower lumbar spine, suspicious of tumor spreading to his skeleton. This clinical picture, and the first immunohistochemical results showing intensive staining of cathepsin K, pointed to dysfunctional activity in osteoclasts, and linked therapeutic choices to autophagy and metabolism. He was referred to surgical resection of the part of his chest wall afflicted by a tumor mass, which was conducted at the University Hospital Oslo in September 2013. Postoperatively, he received irradiation to the tumor bed, 3 Gy per fraction to a total dose of 30 Gy.

Today, there is no commonly accepted treatment recommendation for non-RCC. Pending the final histology report, while having the clinicopathological suspicion of translocational RCC, several therapeutical strategies were discussed. In the light of no available standard treatment for patients with translocational RCC, a mammalian target of rapamycin (mTOR) inhibitor (everolimus 10 mg daily) counteracting phosphorylation of TFEB by mTOR complex 1 (mTORC1) [6] and denosumab (120 mg subcutaneously every fourth week), a monoclonal antibody regulating osteoclast activity by targeting the RANK ligand, were both applied during and after postsurgical radiotherapy. This therapeutic strategy aimed to counteract the dysfunctional signaling effect in catabolism and bone reabsorption as described for TFEB tumors. In addition, the formerly nephrectomized patient had moderately reduced kidney function after the operation, and needed self-catheterization. Often tyrosine kinase inhibitors (TKIs) affect kidney function, supporting an mTOR pharmacological treatment instead of TKI for this patient. Unfortunately, everolimus led to thrombocytopenia and had to be reduced to 7.5 mg per day taken orally. During the next 2 months, the patient’s health deteriorated, and the mTOR-inhibitor therapy was eventually interrupted.

A new series of MRI (Fig. 1) confirmed progression, with multiple additional bony lesions. The patient was treated with a broadly acting TKI, pazopanib (800 mg once daily) as second-line medical treatment, interfering with platelet-derived growth factor (PDGF) and vascular endothelial growth factor (VEGF) pathways. At the same time, neither anti-1-amino-3-18F-fluorocyclobutane-1-carboxylic acid (^18^F-FACBC) positron emission tomography (PET)/CT nor 2-deoxy-2-(^18^F)fluoro-D-glucose (^18^F-FDG) PET/CT scans detected tumor spreading.

Acknowledging the difficulty in identifying a rational therapeutic approach, the patient consented to serial biopsies taken in February and June 2014, preceding the choice of a new treatment due to a major lesion in his sacrum. Both samples were then assessed molecularly by Agilent SureSelect exome capture with Illumina sequencing, and expression profiling using bead arrays from Illumina.

Ribonucleic acid (RNA) extraction from the tumor taken at the first time point was split in two parts and a TissueLyser (Qiagen) was used to disrupt the sample. RNA from both parts was extracted using a GenElute Mammalian Total RNA Miniprep Kit (Sigma-Aldrich), according to the manufacturer’s instructions. DNA was extracted from the tumor taken at the second time point, using the NucleoSpin Tissue Kit (Macherey-Nagel), according to the manufacturer’s protocol. Normal DNA was extracted from ethylenediaminetetraacetic acid (EDTA) blood using the NucleoSpin Blood Midi Kit (Macherey-Nagel), according to the manufacturer’s protocol. Normal and tumor DNA was subjected to whole exome sequencing using the SureSelect whole exome v5 and Illumina sequencing by synthesis technology (HiSeq 2500) following the supplier’s protocol. The resulting FASTQ files were further analyzed using an in-house developed pipeline for somatic event detection. Reads of the tumor and its matched control sample were separately mapped with BWA-MEM [7] to human reference genome (build b37) with an added decoy contig, obtained from [8]. Sample-wise sorting and duplicate marking was performed on the initial alignments with Picard tools [9]. Genome Analysis Toolkit (GATK) tools [10] were subsequently used for two-step local realignment around indels (in this step, both samples were processed together). Each sample’s pair-end read information was then checked for inconsistencies with Picard, and base-quality recalibration was performed by GATK.

Somatic variant calling on the sample pair was done with MuTect [11] (somatic single nucleotide variant, SNV, detection), Strelka [12] (somatic SNV and INDEL detection), DELLY [13] (large-scale variation – deletions, duplications, translocations and inversions – detection) and VarScan2 [14] (somatic copy number variation, CNV, analysis).

GATK tools were used for computing coverage statistics based on the recalibrated alignment files.

Most of the analysis (starting with the local realignment step) was limited to exome regions, defined by Agilent sequencing probes (for further details, see Additional file 1).

One translocation was identified through sequencing, involving positions chr11:65,267,772 and chr6:41,659,234, involving the expected TFEB translocation previously described for this patient.

We identified 23 somatic single SNVs, and three insertion/deletion events. Among these, one mutated gene was located in the tumor necrosis factor (TNF) receptor pathway (MAP3K7, G110V), a pathway previously implicated in clear cell RCC [15]. However, no obvious candidate therapeutic target genes were identified as mutated.

**Fig. 2:**
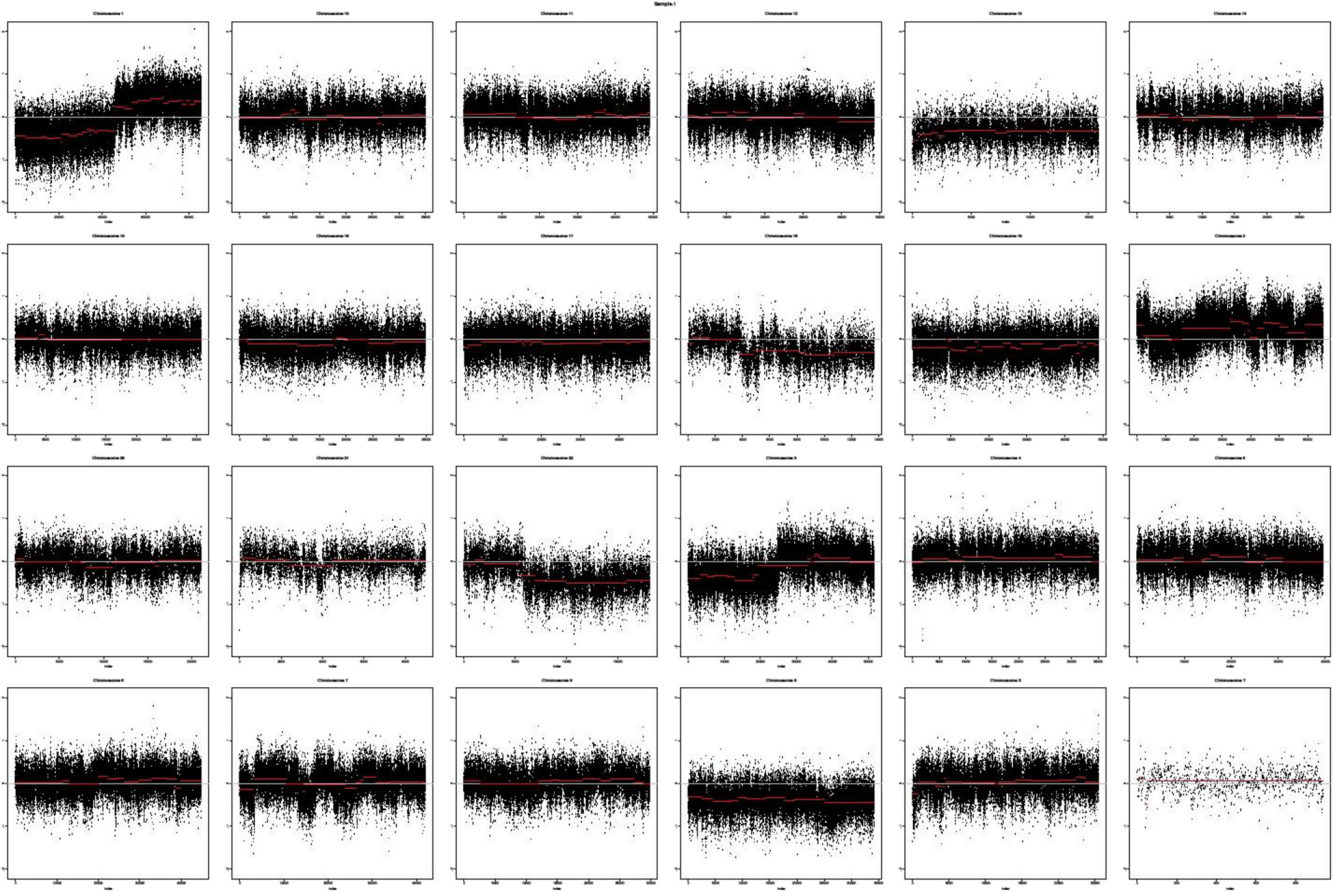
Copy number analysis by chromosome (using VarScan2/DNAcopy)

A DNA copy number plot is shown in Fig. 2. Gain of a region of 1q including the gene *KIF14* has previously been associated with fatal progression, and *KIF14* was among the genes reported to be overexpressed in tumors with 1q [16]. We did identify a somatic mutation for this gene (H849Y), but the tumorigenic potential remains unclear. Other chromosome arm scale events with more or less clear breaks could be seen on chromosomes 1, 3, 18 and 22. Some chromosomes (9, 13, 19) appeared to be generally underrepresented by sequencing reads in the tumor, while some other chromosomes (for example 2 and 7) seemed to be affected by many smaller events.

Transcriptome analysis was performed using the TruSeq Stranded Total RNA with Ribo-Zero Gold from Illumina according to the manufacturer’s instructions. The RNAa-Seq library was sequenced using a HiSeq 2500 with a paired-end 2×100 base pair (bp) approach. The sequencing reads were mapped with TopHat2 [17] to human reference genome/transcriptome (build hg19) and subsequently processed by the Cufflinks2 tools [18] (cufflinks, cuffmerge, cuffdiff) in order to generate a gene-wise fragments per kilobase of transcript per million mapped reads (FPKM) expression (Table 1).

**Table 1:** Somatic mutations detected in tumor

Variant ID	Vartype	Gene	Consequence	Coding change	Depth tumor	AF tumor	Depth normal
chr1_200567369_G_A	snv	*KIF14*	missense_variant	p.H849Y	225	0.227	99
chr1_235909689_G_GCC	INdel	*LYST*	frameshift_variant&feature_elongation	p.A2640fs	575	0.177	255
chr1_43239249_C_T	snv	*C1orf50*	stop_gained	p.R71X	330	0.352	296
chr10_124897147_G_T	snv	*HMX3*	missense_variant	p.G325V	252	0.286	189
chr10_48388199_C_T	snv	*RBP3*	missense_variant	p.M893I	253	0.229	149
chr12_31238059_A_G	snv	*DDX11*	missense_variant	p.R213G	32	0.188	16
chr14_57858330_G_A	snv	*NAA30*	missense_variant	p.E219K	165	0.212	94
chr14_57858331_A_T	snv	*NAA30*	missense_variant	p.E219V	165	0.218	94
chr17_28887148_T_A	snv	*TBC1D29*	missense_variant	p.L9Q	140	0.05	89
chr17_7256853_G_A	snv	*KCTD11*	missense_variant	p.E198K	167	0.192	132
chr18_14542791_C_T	snv	*POTEC*	missense_variant	p.A119T	84	0.083	47
chr19_35842950_G_A	snv	*FFAR1*	missense_variant	p.G166S	564	0.278	358
chr2_202492837_T_A	snv	*TMEM237*	missense_variant	p.N302I	191	0.215	90
chr20_23065590_C_G	snv	*CD93*	missense_variant	p.G414R	410	0.21	230
chr21_45535623_C_A	snv	*PWP2*	missense_variant	p.P220T	130	0.208	96
chr3_108348015_A_G	snv	*DZIP3*	missense_variant	p.T230A	114	0.272	45
chr4_110885598_C_T	snv	*EGF*	stop_gained	p.R494X	402	0.274	260
chr4_155298451_T_A	snv	*DCHS2*	missense_variant	p.E127V	225	0.164	113
chr5_178771091_C_A	snv	*ADAMTS2*	missense_variant	p.V71L	333	0.258	194
chr5_65105904_TC_T	inDEL	*NLN*	frameshift_variant&feature_truncation	p.Q586fs	333	0.168	200
chr6_136589425_G_T	snv	*BCLAF1*	missense_variant	p.P758T	123	0.089	35
chr6_33406615_C_G	snv	*SYNGAP1*	missense_variant	p.T532R	345	0.22	241
chr6_91271355_C_A	snv	*MAP3K7*	missense_variant	p.G110V	558	0.246	305
chr7_143096972_G_T	snv	*EPHA1*	missense_variant	p.R203S	222	0.212	153
chr9_139571036_C_T	snv	*AGPAT2*	splice_donor_variant	exon5:c.588 + 1G > A	83	0.337	103

*snv* single nucleotide variant; *INdel* insertion of the deletion of the bases

**Fig. 3:**
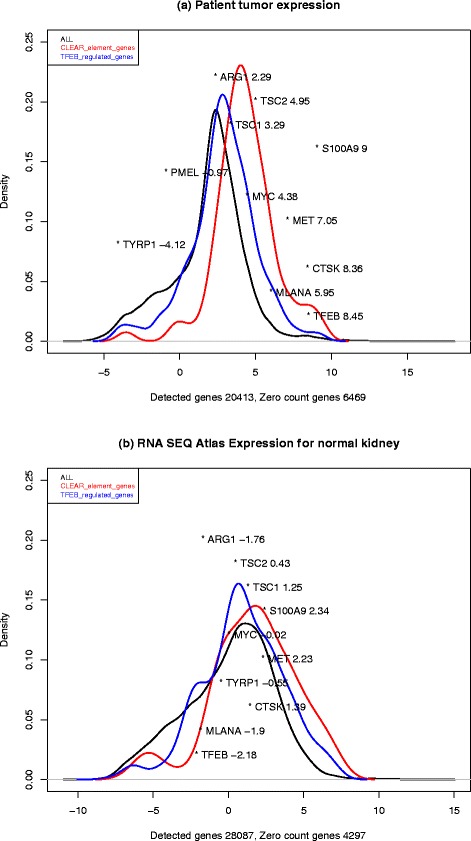
Density plots showing the distributions of ribonucleic acid expression levels for all genes detected in the tumor (*black curve*; **a**) and the RNA-Seq Atlas (**b**). Two subsets of genes are also shown: the *blue curve* demonstrates the genes previously reported to be induced in cells where transcription factor EB is transiently overexpressed, and the *red curve* illustrates the coordinated lysosomal expression and regulation motif (GTCACGTGAC) which overlaps the E-box motif (CANNTG) to which transcription factor EB also binds. The x-values represent the log 2 values of fragments per kilobase of transcript per million mapped reads. Also shown are specific expression levels for genes discussed in the text. *PMEL* was not found in the RNA-Seq Atlas. The y-values for these genes bear no meaning, other than to avoid overlap of names

A plot of normalized transcript counts is shown in Fig. 3a. In order to estimate upregulation of transcription compared to a normal kidney, we obtained transcript counts from the RNA-Seq Atlas [19] (Fig. 3b). In order to evaluate whether there was an expression effect of TFEB being overexpressed, we examined whether genes that previously had been reported as upregulated in transiently TFEB-overexpressed HeLa cells were also affected in the tumor (Fig. 3a, TFEB curve). A marked curve shift towards higher transcript levels, compared to the full list of genes, was observed, indicating that the high TFEB level observed in the tumor also increased expression of the reported target genes. We also examined whether a previously reported gene set for coordinated lysosomal expression and regulation (CLEAR) was upregulated in the tumor, and this could also be observed. Moreover, TFEB expression is very high in the tumor, compared to TFEB expression levels observed otherwise in normal kidney tissues [20, 21].

The transcriptional profile confirmed the clinical suspicion of highly upregulated macro-autophagy and dysfunctional pathway activity in c-MET, MAPK, TSC2 and S100A9, and downregulation of mTOR, as previously reported relevant for this type of tumor (Fig. 3). Treatment with an autophagy-inhibiting agent, hydrochloroquine 200 mg twice a day, was therefore started and subsequently increased to 400 mg twice a day, but stopped when progression was observed in July 2014.

Unfortunately, 5 months after the start of pazopanib, the CT and MRI evaluation confirmed progression, and the treatment was switched to another broad-acting TKI, sunitinib dose 37.5 mg per day, as third-line therapy.

**Fig. 4:**
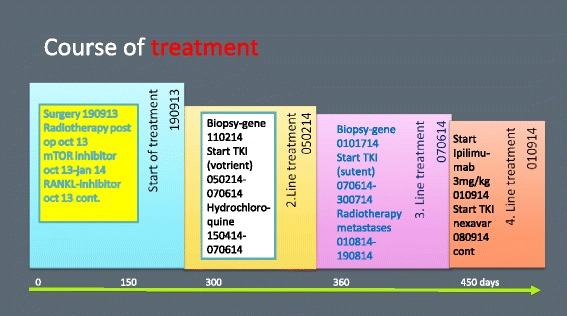
The course of treatment

In the wake of the genetic results and the approval of checkpoint inhibitor therapy, a second course of palliative radiotherapy was planned in late July 2014 (see Fig. 4).

The second biopsy results gained from DNA pointed to enhanced autophagy gene signature and MAPK and AKT pathways downstream. On histological examination, cathepsin K and Melan-A were highly positive. Due to local pain in the patient’s chest wall, a second course of three-dimensional CT conformal radiotherapy encompassing pleural metastases (3 Gy per fraction, 5 days per week) was planned 4 weeks later, and sunitinib treatment was halted prior to radiotherapy. Acute pleural bleeding confirmed with a CT scan showed tumor growth after 8 fractions of radiotherapy, and further irradiation was abolished. Sunitinib treatment was discontinued.

**Fig. 5:**
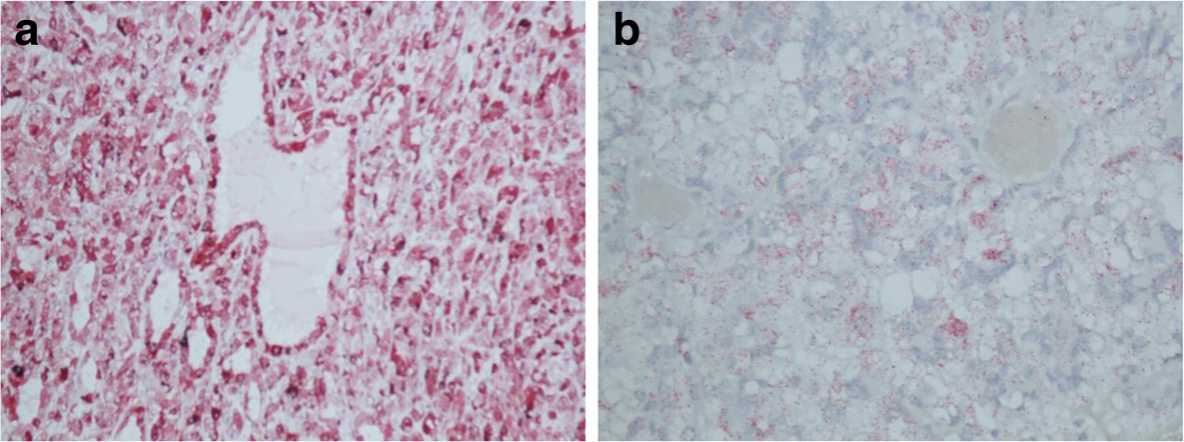
**a** Primary tumor with positive Melan-A staining. **b** Metastasis with positive Melan-A staining

In view of the young age of the patient and the specific staining for Melan-A expressing tumor cells [22] (Fig. 5), he was finally accepted for ipilimumab, targeting CTLA-4, activating the immune system, combined with third-line sorafenib treatment, a kinase inhibitor used in the treatment of renal cancer. Ipilimumab was withdrawn rapidly, due to controversies around combined treatment in the physicians group, exaggerated by temporary health deterioration of the patient. At last follow-up, 16 months after the diagnosis of translocational RCC, the patient’s sorafenib treatment stopped due to disease progression and he died shortly afterwards.

Attempts of cell culture of excess cell material from the bone biopsy in medium were unsuccessful.

## Discussion

A subclass of renal tumors, translocation renal tumors, involves somatic translocations of transcription factors that are members of the *MITF* family, essentially deregulating gene expression control. Translocations involving the *TFEB* are less common than transcription factor E3 (TFE3), with only 30 confirmed cases reported in the literature [23]. Here we describe the therapeutic despair which comes along when treating such a fatal cancer, and an attempt to apply molecular guidance for treatment of this disease. To the best of our knowledge, it is the first such study of the clinical course of TFEB RCC combined with genetic elucidation in a young man with metastatic disease. There is a paucity of comprehensive reports on the clinicogenetic findings in translocation-driven RCC, but recently the clinicopathological manifestations in TFEB have been acknowledged, contributing to the correct diagnosis [23]. The sequencing-based approaches added some important clues to the understanding of this entity in our patient, primarily from expression profiling, as a few mutations that can potentially serve as a drug target could be identified. The main finding in our case was the upregulated activity in autophagy. Dysfunctional autophagy activity guided us in our search for interpretation of the results showing activity in genes and transcript expression in self-destruction [24, 25]. The *TFEB* gene mutation correlated clinically with proliferation and invasion of bony structures, liver and pleural manifestations. The tumor exhibited an extremely aggressive clinical behavior during radiotherapy and was non-responsive to mTOR inhibitor, and moreover showed only modest response to a variety of medical interventions and TKIs. In addition, patients with von Hippel–Lindau (VHL) syndrome can develop clear cell RCC and a subgroup of such patients may have similarities with those described in the transcriptome of our study [26].

The role of *MITF* family transcription factors in renal cell transformation to mutant cancer cell has yet to be elucidated in depth. However, the transcription of TFEB is acknowledged as the master regulator of the mTOR nutrients sensing machinery, in a hypoxia-inducible transcription factor (HIF) independent manner [27]. The intriguing overlap between the more common phenotype of RCC to translocation-driven RCC constitutes the metabolic disarrangement leading to dysfunctional activity in these cells and, as we propose in our case, to uncontrolled autophagy.

In our data, the TFEB driver mutation favored enhanced osteoclast activity, and upregulated cathepsin K was confirmed in several biopsies, and promoting a gene signature connected to MAPK and c-MET signal pathways. It is known that TFEB, the master regulator of lysosome biogenesis, controls transcription of target genes closely related to lysosomal structure and function, including hydrolases. Our findings in the DNA motif demonstrated upregulation of genes previously reported to be induced in cells where TFEB was overexpressed. The same signature overlapped with the CLEAR motif in RNA samples. Tsuda *et al*. showed recently that mTORC1 is a key upstream kinase that directly phosphorylates TFEB and inhibits its activity [28]. In addition, nuclear TFEB translocation may re-establish autophagy, enforcing metabolic activity in cancer cells normally seen in periods of starvation conducted by the autophagy program. Again, the gene signature showed increased expression of autophagy-related genes, supporting our assumption.

Second, in our search for involved signaling pathways and oncologic drivers, we found that c-MET RNA was increased in our obtained biopsies. Our patient had bone lesions early in the course of his metastatic disease, which is in line with the c-MET hyperactivity seen in germline mutations of hereditary papillary RCC and bone metastases of RCC. Moreover, patients with upregulation of lysosomes in osteoclasts might benefit from c-MET inhibitors, such as cabozantinib, where the c-MET signal pathway is stimulated by TFEB to trigger lysosomal acidification and degradation of the extracellular bone microenvironment supporting the activity of osteoclasts [29]. In our case, the metastasis stemmed from the rib, and the first signs of disseminated spread were localized to the spine. We tried to counteract the possibility of tumor-related autophagy with hydrochloroquine. This was an unproven experimental off-label approach, but has been tried in other solid tumors before. Unfortunately, we did not have access to c-MET inhibitors, which would have been a more targeted attempt supported by the gene expression findings from the biopsies.

Some interesting similarities, but also striking differences, to clear cell RCC were seen. In clear cell RCC, mutations in the *VHL* gene lead to upregulation of mTOR and glycolysis. In TFEB RCC, mechanisms different from aerobic glycolysis may be active. One possible major difference is the involvement of c-MYC, which has been linked to HIF-1 metabolism and glutaminolysis [30]. One other interesting feature in our patient with autophagy addiction points to the use of the amino acid arginine, and an increased arginase activity was seen, which also could be clinically exploited. The often related metabolic shift in energy rescue seen in clear cell RCC was not apparent in our case. This may have consequences for imaging, in that ^18^F-FDG PET may not be suitable to detect disease activity. Previously, baseline high ^18^F-FDG PET uptake and increased number of positive lesions have been associated with prognostic information in patients receiving sunitinib [31]. As seen in our patient, the hybrid PET/CT combined with ^18^F-FDG or ^18^F-FACBC as radiotracers could not convey valuable information about the location or activity of metastases. However, the possible addiction for glutamine and/or arginine and possible dysfunctional metabolism should be tested with novel amino acid radiotracers in these patients. In the wake of specific TFEB RCC radiotracers, it seems that the first choice of standard radiological diagnosis in this tumor is MRI. MRI assessment conceived bone and soft tissue lesions and was reliable to address the efficacy of target therapy.

## Conclusions

The clinical features with initially osteolytic bony lesions in our patient, together with the recognized autophagic genetic fingerprint supervised our therapeutic approach, representing a first step into precision medicine for this patient group.

The efficacy of TKIs and mTOR inhibitors appears limited in translocational RCC. Treatment duration in those patients may last when intrinsic patterns of resistance to a particular agent could be obtained. To do so, serial biopsies of metastatic lesions were sampled in our patient. We found a clear molecular link to activated autophagy and osteoclast enhancement, but poor response to existing targeted therapy. Of note, a possible durable effect by ipilimumab in combination with a TKI presumably stimulating the immune system and this observation deserves further investigation. Despite the limited therapeutic benefits achieved in our case, clarifying the essential genetic make-up may portend the future treatment in this rare disease.

## Consent

Written informed consent was obtained from the patient for research purposes when he was still alive. A copy of the written consent is available for review by the Editor-in-Chief of this journal.

## Additional file

Additional file 1: Reference data DNA/RNA analysis and individual pipeline steps DNA/RNA (DOCX 22 kb)
