# Supplementary material for: Translocational renal cell carcinoma (t(6;11)(p21;q12) with transcription factor EB (TFEB) amplification and an integrated precision approach: a case report
Source: J Med Case Rep. 2015 Dec 9;9:281. doi: 10.1186/s13256-015-0749-7 (PMC4674949; doi:10.1186/s13256-015-0749-7)
Supplement: Additional file 1: — Reference data DNA/RNA analysis and individual pipeline steps DNA/RNA (DOCX 22 kb) [file 13256_2015_749_MOESM1_ESM.docx]

Additional file 1

**Reference data DNA analysis**:

- human reference genome build b37 with an added decoy contig (from GATK bundle 2.8 – b37 [6]);
- indel collection: ‘1000G_phase1.indels.b37.vcf’ (from GATK bundle 2.8 – b37);
- indel collection: ‘Mills_and_1000G_gold_standard.indels.b37.vcf’ (from GATK bundle 2.8 – b37);
- dbSNP (release 138) variants collection: ‘dbsnp_138.b37.vcf’ (from GATK bundle 2.8 – b37);
- COSMIC [7] (release 64) variants collection: ‘cosmic_v64_sorted_b37.vcf’;
- list of sequencing-probe capture-target regions extended by 250 bp in both directions: ‘agilent_exome_covered_250bp_extended.intervals’;
- list of sequencing-probe capture-target regions: ‘agilent_exome_covered.intervals’.

**Reference data RNA analysis**:

- human reference genome build hg19, with the corresponding Bowtie2 index and transcriptome annotation (versions bundled together in iGenomes package from June 2012 were used).

**Individual pipeline steps DNA analysis:**

1. <input: lane-wise fastq.gz files generated from sequencer output during demultiplexing>

Mapping of each sample’s input reads with BWA mem (version 0.7.8-r455). Performed lane-wise.

The following optional parameters were used:

- -t 20
- -T 0
- -I 200,75
- -R <sample-specific read group information>
- -M

1. <input: lane-wise SAM output of step 1)>

SAM file coordinate sorting and SAM to BAM format conversion. Performed lane-wise for each sample with Picard’s ‘SortSam’ tool (Picard tools version 1.84).

The following optional parameters were used:

- SORT_ORDER=coordinate
- VALIDATION_STRINGENCY=LENIENT
- CREATE_INDEX=true

1. <input: lane-wise BAM output of step 2)>

Merging of lane-wise alignments and marking of duplicate reads, performed individually for each sample with Picard’s ‘MarkDuplicates’ tool (Picard tools version 1.84). Subsequent indexing was performed with Samtools’ “index” command (Samtools version 0.1.18). No optional parameters were used in this step.

1. <input: a matched pair of BAM files generated in step 3)>

2-step local realignment around indels. Performed jointly on both samples with GATK’s ‘RealignerTargetCreator’ and ‘IndelRealigner’ tools (version 2.3-9-ge5ebf34).

The following optional parameters were used with the ‘RealignerTargetCreator’ tool:

- -known 1000G_phase1.indels.b37.vcf
- -known Mills_and_1000G_gold_standard.indels.b37.vcf
- -L agilent_exome_covered_250bp_extended.intervals
- -nt 24

The following optional parameters were used with the ‘IndelRealigner’ tool:

- -known 1000G_phase1.indels.b37.vcf
- -known Mills_and_1000G_gold_standard.indels.b37.vcf
- -L agilent_exome_covered_250bp_extended.intervals
- -targetIntervals <output of RealignerTargetCreator’s run for the same sample pair>
- -nWayOut <output file name suffix>

1. <input: sample-wise BAM output of step 4)>

Application of Picard’s ‘FixMateInformation’ tool to each sample (Picard tools version 1.84).

The following optional parameters were used:

- SORT_ORDER=coordinate
- VALIDATION_STRINGENCY=LENIENT
- CREATE_INDEX=true

1. <input: sample-wise BAM output of step 5)>

2-step base-quality recalibration. Performed for each sample with GATK’s ‘BaseRecalibrator’ and ‘PrintReads’ tools (version 2.3-9-ge5ebf34).

The following optional parameters were used with the ‘BaseRecalibrator’ tool:

- -cov ContextCovariate
- -cov CycleCovariate
- -knownSites dbsnp_138.b37.vcf
- -knownSites 1000G_phase1.indels.b37.vcf
- -knownSites Mills_and_1000G_gold_standard.indels.b37.vcf
- -plots <pre-recalibration covariate-plots file for given sample>
- -nct 8

The following optional parameters were used with the ‘PrintReads’ tool:

- -BQSR <output of BaseRecalibrator’s run for the same sample>
- -nct 8

1. <input: a matched pair of BAM files generated in step 6)>

Somatic SNV calling, performed on the sample pair with MuTect (version 1.1.4).

The following optional parameters were used:

- --cosmic cosmic_v64_sorted_b37.vcf
- --dbsnp dbsnp_138.b37.vcf
- -L agilent_exome_covered_250bp_extended.intervals
- --enable_extended_output

1. <input: a corresponding pair of BAM files generated in step 6)>

Somatic SNV and INDEL calling, performed on the sample pair with Strelka (version 1.0.13).

Template configuration file “strelka_config_bwa_default.ini” was used with the following changes:

- value of parameter “isSkipDepthFilters” was set to 1
- value of parameter “isWriteRealignedBam” was set to 1

The make command which starts Strelka analysis was run with option “-j 20”.

1. <input: a corresponding pair of BAM files generated in step 6)>

Copy number variation analysis was performed with Samtools (version 0.1.18), Varscan 2 (version 2.3.5) and R-package DNAcopy (R version 3.0.3, DNAcopy version 1.40.0). The following steps were performed:

a) Samtools ‘mpileup’ command was used for creating a joint mpileup file for the input sample pair (the normal sample alignment file being provided before the matched tumor sample alignment file). Optional parameter “-B” was used.

b) Normal/tumor read-count ratio was determined with help of Samtools ‘view’ command. Only reads with the following FLAG bits unset were included in the counting: 0x4, 0x100, 0x400 (i.e. unmapped and duplicate reads were excluded, only primary alignment lines were considered).

c) VarScan2 ‘copynumber’ command was run on the mpileup file from step 9-a), with ratio value determined in step 9-b) serving as the only optional parameter.

d) VarScan2 ‘copycaller’ was applied on result of step 9-c).

e) R-package DNAcopy was used for segmentation of output provided by step 9-d). Please see the R-code section below for details.

1. <input: a matching pair of BAM files generated in step 3)>

Large-scale variation detection (including discovery of duplications, deletions, inversions and translocations) was performed with Delly (version 0.5.5) jointly on both samples. The “-x” option intended for exclusion of specified genomic regions was utilized (the standard human reference region exclusion list – a part of the Delly source-code package – was used).

Somatic filtering was subsequently performed with script “somaticFilter.py” (a part of the Delly source-code package) for each variant type (options “-f”, “-m 50” and “-a 0.2” were used).

1. <input: a corresponding pair of BAM files generated in step 6)>

Coverage statistics were computed for each sample by GATK’s ‘DepthOfCoverage’ tool (version 2.3-9-ge5ebf34).

The following optional parameters were used:

- -omitBaseOutput
- -L agilent_exome_covered.intervals
- -ct X

The “-ct X” option was used multiple times, with X ranging between 5 and 100 (all multiples of 5 within that range were used).

**Individual pipeline steps RNA analysis:**

1. <input: the initial fastq files >

Mapping with TopHat2 (version 2.0.11), using Bowtie2 [3] (version 2.2.1) and Samtools (version 0.1.18).

The following optional parameters were used:

- -p 8
- -G <iGenomes hg19 transcriptome annotation>

1. <input: output of step 1)>

Cufflinks2 (version 2.1.1) analysis of the sample transcriptome.

The following optional parameter was used:

- -p 8

1. <main input: output of step 2)>

Merging of the iGenomes transcriptome annotation with transcripts detected in step 2); performed with cuffmerge (part of the Cufflinks tools, version 2.1.1).

The following optional parameter was used:

- -p 8
- -g <iGenomes hg19 transcriptome annotation>
- -s <iGenomes hg19 genome>

1. <main input: output of steps 1) and 3)>

Differential expression analysis by cuffdiff (part of the Cufflinks tools, version 2.1.1). The patient sample was compared to itself, merely for generating the gene-wise FPKM expression values in a standard cuffdiff output format.

The following optional parameters were used:

- -p 8
- -b <iGenomes hg19 genome>
- -u <output of step 3)>
- -L P,U

One of the result files (“genes.fpkm_tracking”) was used by Vegard in his analysis.

**R-code used during CNV analysis**

options(expressions=20000);

library('DNAcopy');

cn <- read.table(<output of VarScan2 copycaller for a given sample pair>, header=T);

CNA.object <- CNA( genomdat = cn[,7], chrom = cn[,1], maploc = cn[,2], data.type = 'logratio');

CNA.smoothed <- smooth.CNA(CNA.object);

segs <- segment(CNA.smoothed, verbose=0, min.width=2);

segs2 = segs$output;

write.table(segs2[,2:6], file='<sample-pair prefix>.copynumber.called.smoothed', row.names=F, col.names=T, quote=F, sep='\t');

segsp = segments.p(segs);

write.table(segsp, file='<sample-pair prefix>.copynumber.valued', row.names=F, col.names=T, quote=F, sep='\t');

pdf('<sample-pair prefix>.copynumber.by_chrom.relative_pos.visual.pdf', 45, 30);

plot(segs, plot.type='samplebychrom');

dev.off();

pdf('<sample-pair prefix>.copynumber.by_chrom.exact_pos.visual.pdf', 45, 30);

plot(segs, plot.type='samplebychrom', xmaploc=TRUE);

dev.off();

pdf('<sample-pair prefix>.copynumber.genome.visual.pdf', 45, 45);

plotSample(segs);

dev.off();

segs <- segment(CNA.smoothed, verbose=0, min.width=2, undo.splits='sdundo');

segs2 = segs$output;

write.table(segs2[,2:6], file='<sample-pair prefix>.copynumber.smoothed_sdundo', row.names=F, col.names=T, quote=F, sep='\t');

segsp = segments.p(segs);

write.table(segsp, file='<sample-pair prefix>.copynumber.valued_sdundo', row.names=F, col.names=T, quote=F, sep='\t');

pdf('<sample-pair prefix>.copynumber.by_chrom.relative_pos.visual_sdundo.pdf', 45, 30);

plot(segs, plot.type='samplebychrom');

dev.off();

pdf('<sample-pair prefix>.copynumber.by_chrom.exact_pos.visual_sdundo.pdf', 45, 30);

plot(segs, plot.type='samplebychrom', xmaploc=TRUE);

dev.off();

pdf('<sample-pair prefix>.copynumber.genome.visual_sdundo.pdf', 45, 45);

plotSample(segs);

dev.off();
